# Unmasking Sapovirus: Chronic Enteritis in an Immunocompromised Adult With Hypogammaglobulinemia

**DOI:** 10.7759/cureus.90064

**Published:** 2025-08-14

**Authors:** Alan Wang, John Greene

**Affiliations:** 1 Osteopathic Medicine, Nova Southeastern University Dr. Kiran C. Patel College of Osteopathic Medicine, Clearwater, USA; 2 Infectious Diseases, Moffitt Cancer Center, Tampa, USA

**Keywords:** chronic diarrhea, chronic sapovirus enteritis, immunocompromised, nitazoxanide, viral gastroenteritis

## Abstract

Sapovirus is a recognized cause of acute viral gastroenteritis, predominantly affecting children and older adults. While typically self-limiting in immunocompetent individuals, sapovirus can result in persistent, severe gastrointestinal illness in immunocompromised patients. Chronic sapovirus enteritis remains underrecognized and lacks standardized treatment guidelines. We report the case of a 69-year-old woman with multiple myeloma and profound hypogammaglobulinemia secondary to chimeric antigen receptor (CAR) T-cell therapy who presented with a 10-month history of chronic watery diarrhea. Imaging revealed features of chronic enteritis, and stool polymerase chain reaction (PCR) identified persistent sapovirus shedding. Endoscopic biopsy showed epithelial apoptosis with histologic features mimicking grade 1 graft-versus-host disease (GVHD) in the absence of prior transplantation. The patient failed to respond to corticosteroids and intravenous immunoglobulin (IVIG). She was subsequently treated with nitazoxanide, resulting in marked clinical improvement, weight gain, and complete clearance of viral shedding at follow-up. She was discharged home on a regimen of monthly IVIG and pegfilgrastim for immune support. This case highlights the challenges in diagnosing and managing chronic sapovirus enteritis in immunocompromised hosts. Impaired mucosal immunity, particularly immunoglobulin A (IgA) deficiency, likely contributes to viral persistence. Histologic findings may mimic GVHD-like injury, further complicating diagnosis. While no Food and Drug Administration (FDA)-approved treatments exist, therapies such as nitazoxanide and immunoglobulin may offer benefit; however, these observations remain exploratory and require further study in larger cohorts.

## Introduction

Sapovirus is a non-enveloped virus in the Caliciviridae family, which also includes norovirus and other causes of gastroenteritis in humans and animals. Its genetic material is single-stranded, positive-sense RNA (+ssRNA), meaning it can be directly used by the host cell to synthesize viral proteins and initiate replication. Sapovirus is a significant cause of acute gastroenteritis worldwide, ranking second only to norovirus in prevalence [1,2]. Alongside norovirus, it is a leading viral pathogen responsible for acute gastroenteritis, particularly affecting young children under five and older adults, although it can infect individuals of all ages [3]. Outbreaks often occur in closed or semi-closed settings such as daycare centers, nursing homes, and long-term care facilities, where close contact facilitates person-to-person transmission [4]. Globally, sapovirus is estimated to cause approximately 2.2%-12.7% of all acute gastroenteritis cases [5], although prevalence varies by diagnostic capability, with higher detection rates in high-income countries using molecular testing such as reverse transcription-polymerase chain reaction (RT-PCR).

Transmission occurs primarily via the fecal-oral route, through ingestion of contaminated food or water, or direct contact with infected individuals [3,6]. Clinical manifestations typically include malaise, nausea, vomiting, abdominal cramps, and non-bloody diarrhea, with fever being relatively uncommon [4,5,7]. Emerging evidence suggests potential long-term consequences, particularly in young children. A study identified sapovirus as a risk factor for lower cognitive performance in children under 24 months, raising concerns about its neurodevelopmental impact [8]. This suggests sapovirus may have broader effects beyond gastrointestinal symptoms, potentially influencing neurodevelopment through mechanisms such as inflammation or malnutrition during critical periods of growth. Although sapovirus is typically self-limiting, it has been linked to severe complications in certain cases. A case report documented the first instance of sapovirus-associated colitis complicated by pneumatosis intestinalis and pneumoperitoneum in a pediatric patient, suggesting that under certain conditions, sapovirus may contribute to more serious intestinal pathology [9].

The typical incubation period for sapovirus ranges from 1 to 4 days [1]. In immunocompetent individuals, symptoms are often mild and resolve within 24-48 hours, although viral shedding may persist for up to 3-4 weeks [3,10]. In contrast, immunocompromised individuals, such as premature neonates, solid organ transplant recipients, or patients undergoing chemotherapy, may experience prolonged and severe symptoms. In these populations, infection may result in chronic diarrhea accompanied by weight loss, dehydration, and even acute kidney injury (AKI) due to volume depletion. Extended viral shedding, lasting up to 147 days, has also been reported [1,2,7,8].

Because disease presentation and progression differ between immunocompetent and immunocompromised hosts, management strategies must be tailored. Here, we report a case of chronic sapovirus enteritis in a profoundly immunocompromised patient following chimeric antigen receptor (CAR) T-cell therapy, highlighting diagnostic and therapeutic considerations.

## Case presentation

A 69-year-old Caucasian woman with a past medical history of multiple myeloma and severe hypogammaglobulinemia, characterized by profoundly low immunoglobulin A (IgA) and immunoglobulin M (IgM) levels following CAR T-cell therapy two years earlier, presented with a 10-month history of chronic watery diarrhea. She reported an average of 8-10 watery bowel movements per day, with estimated stool volume exceeding 1.5 L daily, leading to progressive dehydration. She denied blood or mucus in her stool but reported persistent nausea, vomiting, and an unintentional 10-pound weight loss over the preceding three months. She also denied fever, chills, or abdominal pain.

On admission (day 0), her vital signs were within normal limits: temperature, 98.2°F; blood pressure, 128/76 mmHg; heart rate, 98 beats per minute; respiratory rate, 16 breaths per minute; and oxygen saturation, 95% on room air. Her body mass index (BMI) was 26.2 kg/m². Baseline laboratory evaluation revealed mild hyponatremia (Na: 132 mmol/L) and hypokalemia (K: 3.2 mmol/L). A prior bone marrow biopsy revealed no evidence of dysplasia, with a blast count of 0.8%. Serum studies showed a kappa free light chain level of <0.6 mg/L, a lambda free light chain level of 1.34 mg/L, and a kappa/lambda ratio of <0.45. Immunoglobulin levels for both IgA and IgM were <5 mg/dL. A summary of her key laboratory values is provided in Table 1.

**Table 1:** Summary of key laboratory findings on admission. IgA: immunoglobulin A, IgM: immunoglobulin M

Laboratory test	Patient value	Reference range
Sodium	132 mmol/L	136-145 mmol/L
Potassium	3.2 mmol/L	3.5-5.2 mmol/L
Kappa free light chain	<0.6 mg/L	3.3-19.4 mg/L
Lambda free light chain	1.34 mg/L	5.7-26.3 mg/L
Kappa/lambda ratio	<0.45	0.26-1.65
IgA	<5 mg/dL	70-400 mg/dL
IgM	<5 mg/dL	40-230 mg/dL

**Figure 1:**
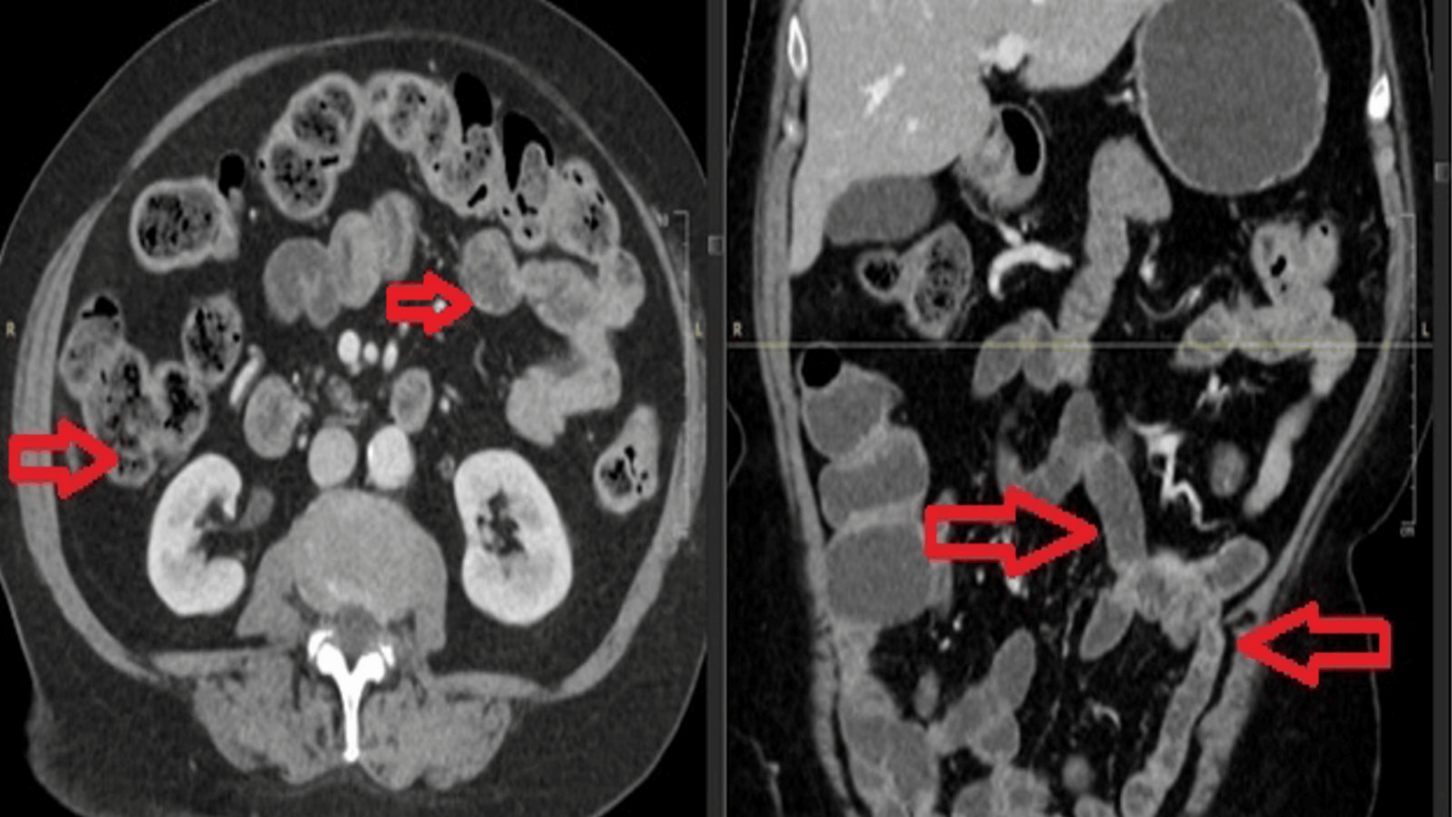
Contrast-enhanced CT images of the abdomen in axial (left) and coronal (right) views demonstrate matted loops of small bowel with wall thickening and increased mucosal enhancement (indicated by arrows). These findings are consistent with chronic enteritis. In the context of an immunocompromised patient with persistent diarrhea, such features raise a strong suspicion for viral enteritis, including sapovirus infection. CT: computed tomography

**Figure 2:**
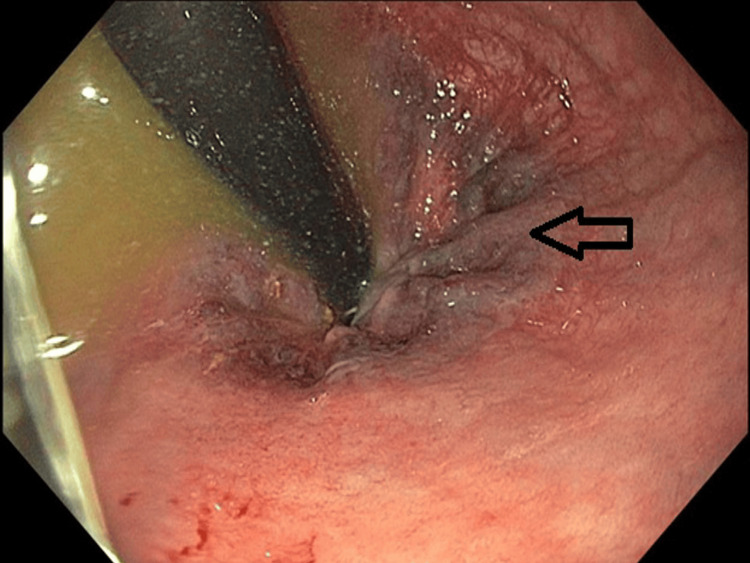
Endoscopic image shows mucosal erythema and friability, with a darkened area of mucosal injury indicated by the arrow. Biopsies obtained from this region revealed increased epithelial apoptosis and histopathologic features resembling grade 1 GVHD, despite the absence of prior allogeneic transplantation. These findings suggest a GVHD-like mucosal injury pattern, possibly triggered by chronic viral enteritis in the setting of profound immunosuppression. GVHD: graft-versus-host disease

Abdominal computed tomography (CT) imaging demonstrated matted loops of bowel, bowel wall thickening, and increased mucosal enhancement, findings consistent with chronic enteritis (Figure 1). Stool studies were negative for common enteric pathogens, including enterotoxigenic* Escherichia coli* (ETEC), *Clostridioides difficile*, *Vibrio* species, and *Cryptosporidium*, but tested positive for sapovirus by PCR on hospital day 3. Repeat testing confirmed persistent viral shedding. Endoscopic biopsies revealed increased epithelial apoptosis, with quantification (normal: <1) showing greater than two apoptotic bodies per crypt in the duodenum and approximately one apoptotic body per crypt in the gastric body and gastric greater curvature. These findings, in the absence of prior allogeneic transplantation, most closely resembled a grade 1 graft-versus-host disease (GVHD)-like mucosal injury pattern related to chronic viral enteritis (Figure 2). Cytomegalovirus (CMV) and adenovirus stains were negative; periodic acid-Schiff (PAS) and acid-fast bacillus (AFB) stains were not performed.

Following CAR T-cell therapy, the patient had been initiated on monthly high-dose intravenous immunoglobulin (IVIG) replacement, with progressive dose escalation over 24 months. Despite this prolonged and intensified regimen, her diarrhea persisted, likely because IVIG does not replenish secretory IgA, a key component of mucosal immunity essential for clearing enteric viral infections. Given the lack of improvement, nitazoxanide 500 mg orally twice daily was initiated on day 12 of admission as a planned 14-day course. She completed the first seven days in the hospital, during which her stool frequency decreased by more than 50%, electrolyte abnormalities resolved, and oral intake improved.

She was discharged home to complete the remaining seven days of nitazoxanide while continuing monthly IVIG infusions and every-three-week pegfilgrastim for immune support, with close follow-up by the infectious diseases team. At her first post-discharge visit, she was diagnosed with small intestinal bacterial overgrowth and treated with rifaximin. One month after discharge, she had gained 3 pounds, diarrhea had resolved, and viral shedding was cleared. Her bone marrow and IgG levels had normalized.

## Discussion

This case underscores the importance of including sapovirus in the differential diagnosis of chronic diarrhea in immunocompromised patients, particularly when routine testing for common pathogens is negative and standard therapies prove ineffective. While management of acute viral gastroenteritis is typically supportive, focused on hydration and electrolyte correction, there is currently no established treatment for chronic sapovirus enteritis, especially in immunocompromised hosts.

The gold standard for diagnosing sapovirus is reverse transcription-polymerase chain reaction (RT-PCR), which amplifies viral RNA with high sensitivity and specificity [3,4]. In recent years, multiplex PCR testing, capable of detecting multiple enteric pathogens from a single stool sample, has become increasingly common in clinical practice, streamlining diagnosis and expediting management decisions [6,11]. In our case, multiplex PCR facilitated rapid exclusion of other common pathogens, such as enterotoxigenic* Escherichia coli *(ETEC), *Clostridioides difficile*, *Vibrio* species, and *Cryptosporidium*, while confirming persistent sapovirus shedding. Since the adoption of these molecular diagnostics, reported sapovirus incidence has risen, not because of an actual increase in infections, but because prior cases were likely underdiagnosed or misattributed to more common viral agents [4]. This underscores the importance of selecting appropriate molecular tests for immunocompromised patients with chronic gastrointestinal symptoms, as early pathogen identification can guide timely, targeted interventions and potentially improve outcomes.

The persistence of sapovirus enteritis in immunocompromised hosts highlights the critical role of the immune system in viral clearance. Specifically, secretory IgA plays a key role in mucosal immunity by activating innate immune responses through the interferon pathway, facilitating viral neutralization and clearance [4,8,12]. An intact host immune response is essential for resolving sapovirus infection and is a major determinant of whether the infection becomes chronic. This is reflected in its epidemiology: chronic sapovirus infections are most commonly seen in premature neonates, the elderly, and individuals with compromised immune function, such as post-transplant recipients. In our patient, chronic sapovirus infection likely resulted from persistent IgA deficiency secondary to prior CAR T-cell therapy, placing her at increased risk for prolonged viral shedding and symptoms. Interestingly, endoscopic biopsy demonstrated features resembling grade 1 GVHD, including increased epithelial apoptosis, despite the absence of allogeneic transplantation. This histologic pattern may represent nonspecific epithelial injury or a GVHD mimic associated with chronic viral enteritis. In our patient, these changes were most consistent with mucosal injury related to chronic inflammation and immune dysregulation, likely driven by the persistent viral infection.

To place this case in context, Table 2 summarizes key published reports of sapovirus infection in immunocompromised patients, highlighting differences in immune status, duration of viral shedding, IgA levels, treatments used, and outcomes. This comparison underscores the variability in clinical course and therapeutic response among such patients, as well as the potential value of targeted therapies such as nitazoxanide and immunoglobulin supplementation.

**Table 2:** Comparison of chronic sapovirus cases in immunocompromised adults. AKI: acute kidney injury, IgA: immunoglobulin A, CAR: chimeric antigen receptor, PCR: polymerase chain reaction, BID: twice a day, IVIG: intravenous immunoglobulin

Study and year	Population	Duration of symptoms	Duration of viral shedding	IgA levels	Immunosuppression	Treatment	Outcome
Roos-Weil et al. (2011) [10]	Kidney transplant	Median: 9 weeks	Median shedding: 289 days (107-581)	Not reported	Various immunosuppressants	Supportive + immunosuppression reduction	81% with AKI; some rejection due to tapering of immunosuppressant
Wright et al. (2020) [6]	Kidney transplant	12 months norovirus + new sapovirus	Not specified	Not reported	Tacrolimus + mycophenolate + prednisone	Immunosuppression reduction + nitazoxanide	Symptom relief; later rejection
Rubio-Mora et al. (2024) [3]	Pediatric kidney transplant	Median: 3 months	Persistent shedding	Low IgA	Tacrolimus	Tacrolimus dose reduction	Gradual improvement
Current case	Multiple myeloma post-CAR T-cell therapy	10 months	Persistent at multiple PCRs	<5 mg/dL	CAR T-cell therapy induced hypogammaglobulinemia	Nitazoxanide 500 mg BID × 14 days + IVIG	Diarrhea improved after 7 days, and weight gain of 3 pounds

This case also highlights how management strategies may differ depending on the immunocompromised state. For example, in post-solid organ transplant recipients, reducing immunosuppressive therapy may aid in viral clearance by restoring immune function. However, this must be balanced carefully against the risk of graft rejection [3,7]. In contrast, our patient's immunodeficiency was therapy-induced and not related to immunosuppressive medication. Given her treatment-related hypogammaglobulinemia, we explored the use of IVIG as a novel strategy to boost mucosal antibody levels and support viral clearance.

One major reason for the lack of standardized treatment for chronic sapovirus enteritis is the limited understanding of the virus's pathogenic mechanisms. Unlike many other viruses, sapovirus cannot be cultured in vitro, which hinders the ability to study its molecular biology and life cycle directly, and the lack of animal models further limits research [12]. As a result, much of what we know about sapovirus is extrapolated from studies of porcine sapovirus, which can be successfully cultured and observed in laboratory settings [13,14]. Because sapovirus and norovirus both belong to the Caliciviridae family, current treatment approaches often mirror those used for norovirus, focusing primarily on supportive care. However, in cases of chronic sapovirus enteritis, especially in immunocompromised patients where symptoms may persist, complications arise, and prolonged viral shedding occurs, there is a pressing need to develop tailored management strategies that account for the unique host-virus dynamics and disease progression.

The management of chronic sapovirus enteritis should differ fundamentally from that of acute infection, as the two follow distinct clinical courses and require different therapeutic strategies. Table 3 summarizes the key differences between acute and chronic disease.

**Table 3:** Comparison of acute versus chronic sapovirus enteritis. CAR: chimeric antigen receptor, AKI: acute kidney injury, RT-PCR: reverse transcription-polymerase chain reaction

Feature	Acute sapovirus enteritis	Chronic sapovirus enteritis
Typical host	Immunocompetent individuals (children, elderly, all ages)	Immunocompromised (e.g., post-transplant, chemotherapy, CAR T-cell therapy)
Incubation period	1-4 days	Same as acute, but symptoms persist
Symptom duration	24-48 hours	≥4 weeks, often persistent or relapsing
Symptoms	Watery diarrhea, nausea, vomiting, malaise, abdominal cramps	Chronic watery diarrhea, weight loss, dehydration, possible AKI
Fever	Rare	Rare, often absent
Viral shedding	3-4 weeks post-symptom resolution	Up to 147 days
Complications	Rare, self-limited	Dehydration, electrolyte imbalance, weight loss, kidney injury, malabsorption
Diagnosis	RT-PCR or multiplex PCR	RT-PCR or multiplex PCR; multiple repeat tests may be needed
Management	Supportive: fluids, electrolytes	Investigational: nitazoxanide, oral or intravenous immunoglobulin, supportive therapy
Prognosis	Excellent	Variable; may resolve with treatment, but can relapse without targeted therapy

Acute sapovirus gastroenteritis is typically self-limiting, and treatment is focused on supportive measures, primarily fluid rehydration and correction of electrolyte imbalances, similar to the management of other viral gastroenteritides [15]. Currently, no Food and Drug Administration (FDA)-approved antiviral therapy exists for sapovirus, and standard care relies on the host's immune system to clear the infection. In contrast, chronic sapovirus enteritis in immunocompromised individuals often warrants a more proactive approach, as persistent infection can lead to prolonged diarrhea, dehydration, malnutrition, weight loss, and other complications. Nitazoxanide, although not FDA-approved for this indication, has shown potential benefit in managing prolonged enteric viral infections [1,3,5,16]. Originally developed as an antiparasitic, it also exhibits broad-spectrum antiviral activity. While its exact mechanism is not fully understood, one proposed explanation, extrapolated from its activity against the hepatitis C virus, is that it may disrupt viral replication by impairing viral protein synthesis through host-regulated pathways such as eIF2α phosphorylation [17]. The rationale for using nitazoxanide in this case stems from its documented efficacy against norovirus, a closely related calicivirus that shares similar replication mechanisms with sapovirus. This biological overlap provides a mechanistic basis for extrapolating its use, particularly in immunocompromised hosts where spontaneous viral clearance is unlikely. Nitazoxanide is generally well tolerated, with mild, self-limiting adverse effects such as abdominal pain and headache [7]. The standard regimen is 500 mg orally twice daily; in our patient, this regimen was prescribed for 14 days. This case underscores the urgent need for targeted antiviral therapies for sapovirus and supports further investigation of nitazoxanide as a potential treatment option.

Polyclonal immunoglobulin therapy is another therapeutic option worth considering in immunocompromised patients, especially those with IgA deficiency, given IgA's important role in mucosal immunity. This approach is particularly relevant in patients like ours, who remain at high risk for chronic infection due to underlying immune dysfunction [16]. Additionally, novel antiviral compounds such as 1CMC and 7DMA, both nucleoside analogs that act by mimicking natural DNA/RNA nucleotides, thereby inhibiting viral RNA replication, have shown in vitro activity against related caliciviruses. While these findings suggest therapeutic potential for sapovirus, clinical trials are still needed to confirm their safety, optimal dosing, and efficacy in human populations [18]. Finally, interferon lambda (IFN-λ), known for its mucosal antiviral activity, is under investigation as a potential treatment for chronic sapovirus infection. Preliminary data suggest that IFN-λ may enhance viral clearance while limiting systemic inflammation, although further clinical research is needed to establish its safety and efficacy in this setting [19].

## Conclusions

Chronic sapovirus enteritis is an underrecognized yet clinically significant cause of persistent diarrhea in immunocompromised individuals. Molecular diagnostics, such as RT-PCR and multiplex PCR, are essential for accurate detection, particularly when conventional testing is negative. This case highlights the importance of early recognition and individualized treatment, especially when standard supportive care fails. In our patient, diarrhea frequency improved and electrolyte balance normalized within six days of targeted therapy, with complete symptom resolution, weight gain, and confirmed clearance of viral shedding at follow-up. While these findings suggest a potential therapeutic role for nitazoxanide and immunoglobulin supplementation, the evidence remains exploratory given the single-patient nature of the report. Further studies are needed to determine whether these interventions consistently improve outcomes in chronic sapovirus infection.
